# Respiratory control in aquatic insects dictates their vulnerability to global warming

**DOI:** 10.1098/rsbl.2013.0473

**Published:** 2013-10-23

**Authors:** Wilco C. E. P. Verberk, David T. Bilton

**Affiliations:** 1 Department of Animal Ecology and Ecophysiology, Institute for Water and Wetland Research, Radboud University, Toernooiveld 1, 6525 ED Nijmegen, The Netherlands; 2 Marine Biology and Ecology Research Centre, School of Marine Science and Engineering, University of Plymouth, Davy Building, Drake Circus, Plymouth PL4 8AA, UK

**Keywords:** climate change, eutrophication, hypoxia, multi stressor, oxygen limitation, respiration physiology

## Abstract

Forecasting species responses to climatic warming requires knowledge of how temperature impacts may be exacerbated by other environmental stressors, hypoxia being a principal example in aquatic systems. Both stressors could interact directly as temperature affects both oxygen bioavailability and ectotherm oxygen demand. Insufficient oxygen has been shown to limit thermal tolerance in several aquatic ectotherms, although, the generality of this mechanism has been challenged for tracheated arthropods. Comparing species pairs spanning four different insect orders, we demonstrate that oxygen can indeed limit thermal tolerance in tracheates. Species that were poor at regulating oxygen uptake were consistently more vulnerable to the synergistic effects of warming and hypoxia, demonstrating the importance of respiratory control in setting thermal tolerance limits.

## 1. Introduction

Climatic warming is currently affecting ecosystems throughout the globe at rates unprecedented in recent geological history [1,2]. Furthermore, ecological impacts of increased temperatures can be exacerbated by other environmental stressors, hypoxia being a principal example in water [3]. There is an urgent need to take such interactions into account to accurately predict biological responses to change, although in most systems, a suitable framework for forecasting responses to multiple stressors remains lacking. Analyses of physiological traits at the organismal level could provide the mechanistic framework required, offering a promising approach to understanding the possible impacts of rapidly changing climate [4,5]. Warming increases ectotherm metabolism and hence oxygen demand, while also increasing the availability of dissolved oxygen via thermally dependent oxygen diffusivity and solubility [6]. Insufficient oxygen has been shown to limit thermal tolerance in several aquatic ectotherms [7–10], as increases in metabolism outweigh increases in availability of dissolved oxygen [6]. This oxygen limitation hypothesis is one of the few paradigms available to understand and predict the relative vulnerability of species to the interactive effects of climate warming and hypoxia; however, its generality has been challenged. In insects, studies to date suggest that hypoxia does not reduce heat tolerance [11,12] and that the oxygen limitation hypothesis may not apply to these tracheated arthropods, which comprise the bulk of biodiversity in inland waters [13]. Here, we revisit the oxygen relations of aquatic ectotherms and demonstrate that oxygen can indeed limit the heat tolerance of aquatic tracheates. Importantly, we resolve the discrepancy between earlier studies on terrestrial arthropods and marine ectotherms by showing that the extent of oxygen limitation at thermal extremes depends on gas exchange mechanism. Our work provides a novel framework, in which the ability to regulate gas exchange (i.e. respiratory control, see §4) is seen to determine the relative vulnerability of taxa to the synergistic effects of global warming and hypoxia.

## 2. Material and methods

Species were collected in southwest England and maintained in the laboratory at 10±1°C in a 12 L : 12 D regime, in aquaria containing artificial pond water, buffered and diluted to reflect the pH and conductivity of field sites. Before recording critical temperatures, all species were acclimated for at least seven days to reduce variability in thermal history [14].

We placed animals in flow-through chambers to assess thermal limits. Water was supplied to these chambers from a 25 l header tank via a tubular counter-current heat exchanger. Water in the header tank was of the same composition as that used to maintain animals and was bubbled with a mixture of O_2_ and N_2_, obtained using a gas-mixing pump (Wösthoff, Bochum, Germany). Individuals were left resting for 1 h at the equilibration temperature of 10°C, after which temperature in the experimental chambers was increased at 0.25°C min^−1^, using a Grant R5 water bath with a GP200 pump unit (Grant Instrument Ltd, Cambridge, UK) connected to the heat exchanger. Temperatures were logged using a HH806 AU digital thermometer (Omega Engineering Inc., Stamford, CT, USA).

Critical temperatures were assessed at normoxia (20 kPa) and hypoxia (5 kPa). Such hypoxic conditions (25% saturation) may seem extreme from the perspective of terrestrial insects but are quite commonly observed in aquatic habitats [15]. In addition, many terrestrial insects live in an essentially aquatic environment for part of their life cycle where they may encounter such hypoxic conditions (e.g. endoparasites, endophytic species, some rotten wood/fruit specialists, etc.). The gas mixture was adjusted 10 min after placing the animals in the small flow-through chambers to allow for gradual exposure to hypoxic conditions during the resting period. The critical maximal temperature, CT_max_, was defined as loss of all movement; reliably scoreable across all taxa. In this state, animals lose their ability to escape from conditions that will lead to their death [16].

Heat tolerance was assessed in pairs of species differing in their ability to regulate gas exchange (i.e. with contrasting degrees of respiratory control). Species pairs were selected from four different orders of aquatic insects (Ephemeroptera, Odonata, Hemiptera and Coleoptera) allowing independent tests. Breathing underwater is a major challenge as less oxygen is dissolved in water and oxygen diffuses much more slowly in water than in air [6]. Consequently, many aquatic ectotherms have evolved a range of respiratory adaptations. By including four different orders, our comparative approach included taxa with different respiratory modes (tegument, gill, plastron and surface exchange). While species from different orders have different capacities for oxygen uptake [15] and thus different levels of heat tolerance at normoxia [10], we selected species carefully to obtain pairwise contrasts in respiratory control within each order: the beetles *Agabus bipustulatus* (Linnaeus, 1767) and *Limnius volckmari* (Panzer, 1793) are surface exchanging and plastron breathing adults, respectively, as are the bugs *Ilyocoris cimicoides* (Linnaeus, 1758) and *Aphelocheirus aestivalis* (Fabricius, 1794). The mayfly and dragonfly nymphs all have gas exchange across their body surface and tracheal gills. The mayfly species differ in their ability to move their gill plates and hence degree of respiratory control, *Ecdyonurus insignis* (Eaton, 1870) being able to beat its gills; *Rhithrogena semicolorata* (Curtis, 1834) having immovable gills. Within the odonates, the dragonfly *Cordulegaster boltonii* (Donovan, 1807) has the rectum modified into a heavily tracheated branchial chamber whose surface acts as a gill. Being able to force water across the respiratory surface through abdominal movement provides greater respiratory control relative to the damselfly *Calopteryx virgo* (Linnaeus 1758), which has instead external gill lamellae. All species are predators/scavengers except the mayflies, which are algal scrapers. Despite dietary differences, taxa were easily sustained during acclimation on chironomid larvae and field substratum.

## 3. Results

**Figure 1.**
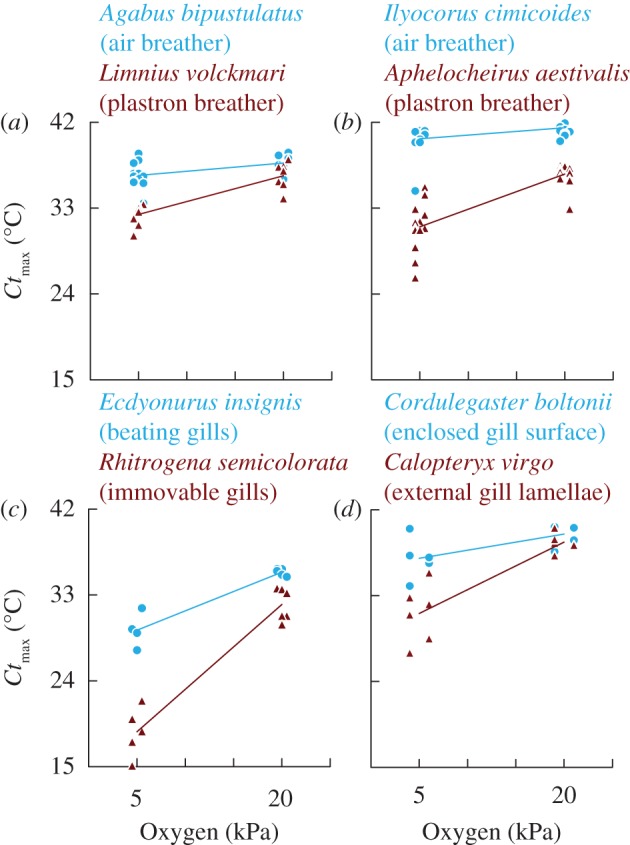
Thermal tolerance limits of four species pairs belonging to (*a*) beetles, (*b*) bugs, (*c*) mayflies and (*d*)dragonflies. In each case, the species with better respiratory control is shown in blue; the other in red. Data points are offset slightly to increase visibility. (Online version in colour.)

In all taxa examined, hypoxia reduced lethal temperatures, but the strength of this effect differed across species (figure 1; electronic supplementary material, table S1). The degree to which species showed such oxygen limitation of thermal tolerance was governed by their ability to regulate oxygen consumption rates—i.e. their respiratory control (ANOVA: interaction between oxygen and respiratory control; *F*_1,160_ = 39.59; *p* = 3.14×10^−9^). As stated above, we compared heat tolerance under normoxia and hypoxia in species pairs which differ in respiratory mode, taken from four different orders of aquatic insects. In each pairwise comparison, across the four orders studied, the species with high respiratory control was consistently less impacted by hypoxia (figure 1).

## 4. Discussion

Understanding how and why respiratory systems are limited in their capacity to supply oxygen to tissues is fundamental to the oxygen limitation hypothesis [8]. Disadvantages associated with maintaining elaborate respiratory structures (e.g. susceptibility of gills to abrasion, higher exposure to toxicants, etc.) may seem small in comparison with having a greater capacity for oxygen uptake (i.e. an overdesigned respiratory system). However, to fully understand the challenges of respiration, we need to think beyond oxygen shortages [17], because breathing oxygen is intrinsically dangerous: while a shortage quickly leads to suffocation, too much is toxic [10,18]. The ability to regulate oxygen consumption rates (i.e. respiratory control) is therefore at a premium; especially in small ectotherms where shifts in external temperatures typically drive metabolic fluctuations. Good respiratory control will enable ectotherms to balance oxygen toxicity against the risk of asphyxiation across a wide range of temperatures; a balancing act which is more challenging for aquatic than aerial gas exchangers [17]. Underwater gas exchange has higher ventilation costs as water is more dense and viscous than air, a problem which is further compounded by lower oxygen content and diffusion rates [6,17].

Our comparative approach spanning four insect orders shows that the extent to which taxa show oxygen limitation at high temperatures is dictated by their degree of respiratory control (figure 1). These comparisons explicitly rule out other potential explanations: the results cannot consistently be related to the architecture of the tracheal system (species with open and closed trachea both exhibited reduced heat tolerance under hypoxia), lower diffusion rate of oxygen in water (an effect of respiratory control is seen with species pairs of mayflies and dragonflies even though all these taxa extract oxygen directly from water) or physiological differences during ontogeny (reduced heat tolerance under hypoxia is seen in both larvae/nymphs and adults). Poor respiratory control quickly results in an organism no longer being able to meet increased oxygen demand at high temperatures. Aquatic ectotherms with poor respiratory control are likely to be thermal specialists, having narrow temperature ranges over which they can balance risks of asphyxiation and oxygen poisoning, and thermal specialists are suggested to be especially vulnerable to warming [4]. The critical thermal maxima of species is strongly linked to their distribution ranges [2,19], making thermal tolerance one of the key traits to examine in conservation physiology [5]. The consistent effect of respiratory control on thermal tolerance provides a novel explanation for the finding that such geographical ranges apparently conform more closely to thermal tolerance limits in marine than terrestrial ectotherms [2]. Because of the tight balance between oxygen risks in aquatic species with weaker respiratory control, they are likely to have more constrained, predictable ranges than terrestrial taxa which are inherently better able to regulate oxygen levels [17].

Oxygen plays a key role in mediating temperature effects, especially in aquatic ectotherms where it explains variation in body size, heat tolerance and geographical range [2,6,10]. Although ectotherms conducting gas exchange in water will have inherent difficulties in controlling oxygen delivery, some species are better able to upregulate oxygen supply under warmer conditions. We have shown that such variation in respiratory control dictates the way aquatic invertebrates respond to heat and to hypoxia (figure 1). In addition to climate warming, eutrophication is a major stressor in both marine and freshwater systems [20,21], its negative impact being largely through resulting hypoxia. Our work shows that oxygen limitation does shape the thermal tolerance of aquatic tracheates, and that respiratory control provides a predictive framework to understand the relative sensitivity of different taxa to these interacting stressors. Aquatic ectotherms which are poor at regulating gas exchange are shown to be especially vulnerable to the multi-stressor effects of increased water temperatures and reduced levels of oxygen. Enhancing water quality, and more specifically improving the degree of oxygenation, is a promising way to improve environmental robustness in the face of climate change and could form a key component of mitigation strategies.

## Data accessibility

All data are publicly available in the electronic supplementary materials.
